# Actovegin in the management of patients after ischemic stroke: A systematic review

**DOI:** 10.1371/journal.pone.0270497

**Published:** 2022-06-30

**Authors:** Philip la Fleur, Ardak Baizhaxynova, Emily Reynen, David Kaunelis, Dinara Galiyeva

**Affiliations:** 1 School of Medicine, Nazarbayev University, Nur-Sultan, Kazakhstan; 2 Department of Critical Care Medicine Queen’s University, Kingston, Ontario, Canada; 3 Canadian Agency for Drugs and Technologies in Health, Ottawa, Canada; Thomas Jefferson University, UNITED STATES

## Abstract

**Background:**

Actovegin is a hemodialysate of calf’s blood and has been used for several decades in the countries of Central Asia, East Asia, Russia and some European countries. It has been used to treat patients with various neurological conditions, vascular disorders, and ischemic stroke.

**Objectives:**

To perform a systematic review to evaluate the effect of Actovegin in patients who have suffered an ischemic stroke.

**Methods:**

A search of MEDLINE, PubMed, Cochrane and Embase was carried out from inception to October 10, 2021 for clinical trials and observational studies with a control group, published in English or Russian.

**Results:**

Of 220 identified unique records, 84 full-text articles were screened, and 5 studies were selected that met the inclusion criteria. This included 4 observational studies with control groups and one randomized, placebo-controlled clinical trial. These studies enrolled a total of 3879 patients of which 720 patients received Actovegin administered intravenously and/or orally for a duration ranging from 10 to 180 days. Because of study heterogeneity, meta-analysis was not performed. No consistent evidence on improved survival, quality of life, neurologic symptoms, activities of daily living or disability was identified. One study showed statistically significant improvements in the Alzheimer’s Disease Assessment Scale, cognitive subscale, extended version (ADAS-cog+) for Actovegin compared with placebo at 6 months but the clinical relevance of this change is uncertain. One study reported a higher incidence of recurrent ischemic stroke, transient ischemic attack or intracerebral hemorrhage in patients taking Actovegin compared to placebo.

**Conclusions:**

The benefits of Actovegin are uncertain and that there is potential risk of harm in patients with stroke. More evidence is needed from rigorously designed clinical trials to justify the role of Actovegin in patients with ischemic stroke.

## Introduction

Despite advances in stroke care in recent decades, stroke remains a significant cause of disability and is the second leading age-standardized cause of death globally [1]. Mortality within the first 30 days after stroke ranges from 16–23 percent and 30-day survivors have an elevated risk of mortality for 15 years after the event. The lifetime risk of ischemic or hemorrhagic stroke in adults is 25% and the risk of ischemic stroke is 18% from the age of 25 years onward [2]. Estimates for the incidence of stroke range from 90–180 per 100,000 population in North America and Western Europe to 241–360 per 100,000 population in East Asia, Eastern Europe and Central Asia [1]. Recent epidemiologic data suggest that the highest incidence of stroke is in East Asia, followed by Eastern Europe and Central Asia [1]. Data from the Global Burden of Ischemic and Hemorrhagic Stroke (GBIHS) study indicate the highest age-standardized mortality rate from ischemic stroke in the world is in Kazakhstan, Central Asia (149–174 deaths per 100,000 person years) [3].

Hyper-acute stroke management has undergone two main advances, intravenous tPA and mechanical thrombectomy, in eligible patients. Despite these advances, ischemic stroke continues to be associated with a high rate of death and disability. One of the mainstays of post stroke management is monitoring and treating potential complications such as adverse events from tPA including angioedema, hemorrhagic transformation of the ischemic stroke, and systemic complications such as infection, arrythmia, heart failure or venous thromboembolism [1]. One key quality indicator in the management of patients after an ischemic stroke is a swallowing assessment to evaluate for dysphagia [2]. Investigating the underlying cause of stroke and optimizing secondary prevention by modifying stroke risk factors both through lifestyle modifications and pharmacotherapy play a crucial role in secondary stroke prevention [2,3]. Management of the chronic phase of stroke care is focused on rehabilitation and maintaining or regaining functional losses [2,3]. Outside of tPA, there are currently no widely accepted pharmacotherapeutic interventions proven to alter the natural history of physical or cognitive impairment which may result from ischemic stroke [4–6].

Actovegin (or Solcoseryl) is a protein-free hemodialysate which is derived from calf blood through an ultrafiltration process [7]. Recent in vitro studies have focused on elucidating anti-oxidant, anti-apoptotic, anti-inflammatory effects and impact on growth factors [8–11]. Hypotheses of therapeutic and neuroprotective effects have been proposed including putative clinical benefits in the context of peripheral and cerebral blood circulation, dermatologic and wound applications, diabetic neuropathy and stroke [12]. Ergogenic effects in athletes have also been explored but no clear benefit has been demonstrated in sporting context [9].

Actovegin was first marketed in 1976 in Germany. It was produced by Nycomed GmbH, Austria, until Nycomed was taken over in 2015 by Takeda Pharmaceutical Ltd Japan [13]. Actovegin has not been marketed in North America but has been promoted and sold as a therapeutic product in East Asia, Austria, Russia, The Commonwealth of Independent States and some eastern European countries.

Recent clinical trials of Actovegin have included populations with diabetic neuropathy, peripheral artery occlusive disease and stroke [14]. Recent epidemiologic data suggest that the highest incidence of stroke is in East Asia, followed by Eastern Europe and Central Asia [1]. These geographic areas correspond with the jurisdictions in which Actovegin has had significant usage. There have been a wide range of interpretations of the evidence for Actovegin ranging from nutritive effects, to placebo effect, to claims of clinically meaningful benefit in the context of stroke despite the fact that the active compound of Actovegin has not been identified[12,15–17]. Clinical guidelines for the treatment of stroke in these geographical areas have included Actovegin in the past, but do not currently include it. The linkage between the emerging evidence on Actovegin, its inclusion in regional guidelines, and its usage in clinical practice is difficult to discern. This systematic review was performed to investigate the amount and strength of evidence to confirm or refute a role for Actovegin in patients with ischemic stroke.

## Methods

### Study question and inclusion criteria

The study protocol was developed a priori by the co-authors based on the population, intervention, comparator, outcome and study design framework. The systematic review was designed to address the question: “Based on controlled studies, does administration of Actovegin result in improved clinical outcomes compared with other pharmacological interventions or placebo in patients with ischemic stroke?” The population of interest was adults with a recent diagnosis of ischemic stroke. The intervention of interest was treatment with Actovegin via any route of administration (oral, intravenous, topical). There must have been a comparator group with a different pharmacological intervention, no treatment or placebo. Controlled trials that did not allow the effects of Actovegin to be isolated were excluded. For example, a two-arm study that used drug A + Actovegin versus drug B, would not allow for an assessment of the effects of Actovegin and would be excluded. Outcomes of interest for this systematic review were survival, cognition, neurologic symptoms, disability, functional impairment, quality of life measured by validated scales and adverse events. Randomized clinical trials were of primary interest, but it was expected that the number of studies would be low and therefore non-randomized trials were accepted. Blinded, open-label, prospective and retrospective studies were permitted but studies without a control group were excluded. Studies available only in abstract format without a full publication were excluded. Our team included bilingual researchers and therefore studies in English or Russian were included.

### Literature search

The literature search strategy team included an experienced information specialist (DK) who designed the electronic literature search to identify relevant evidence. Ovid MEDLINE (1946 to October 10, 2021), PubMed, Cochrane Central Register of Controlled Trials and Ovid Embase (1947 to October 10, 2021) were searched to seek relevant citations. Reference lists of related and retrieved papers were reviewed for additional citations. In addition, the manufacturer of Actovegin was contacted and invited to contribute relevant articles. Clinicaltrials.gov was searched for relevant studies. No language or publication date restrictions were used. The full search strategy is provided in the S1 Table.

### Study screening and selection

Two reviewers (AB, PL or DG) screened citations that were retrieved from the literature search. After reviewing the study abstracts, full text articles were obtained and both reviewers agreed on a final inclusion list.

### Data collection and quality assessment

Data collection from the included studies was performed by two reviewers (AB, PL) using a template designed a priori that included year of publication, study design, length of follow up period, number of participants, descriptions of the intervention and control regimens (dose, route of administration, duration of therapy), characteristics of participants, primary and secondary outcomes, and harms data. Quality assessment was performed using two tools for randomized clinical trials: the Jadad scale and the Cochrane Risk of Bias tool [18,19]. The Newcastle-Ottawa scale was used for observational cohort or case control studies [20]. Two reviewers (AB, PL) applied the scales to each included study.

### Data analysis

We planned to meta-analyze the data if outcomes were reported with sufficient homogeneity. The data did not allow for formal testing of heterogeneity, but there was heterogeneity with respect to study design and study outcomes. Therefore, the authors decided that there were important differences among the studies and meta-analysis was inappropriate. A narrative approach was used to summarize study results.

## Results

### Eligible studies

**Fig 1:**
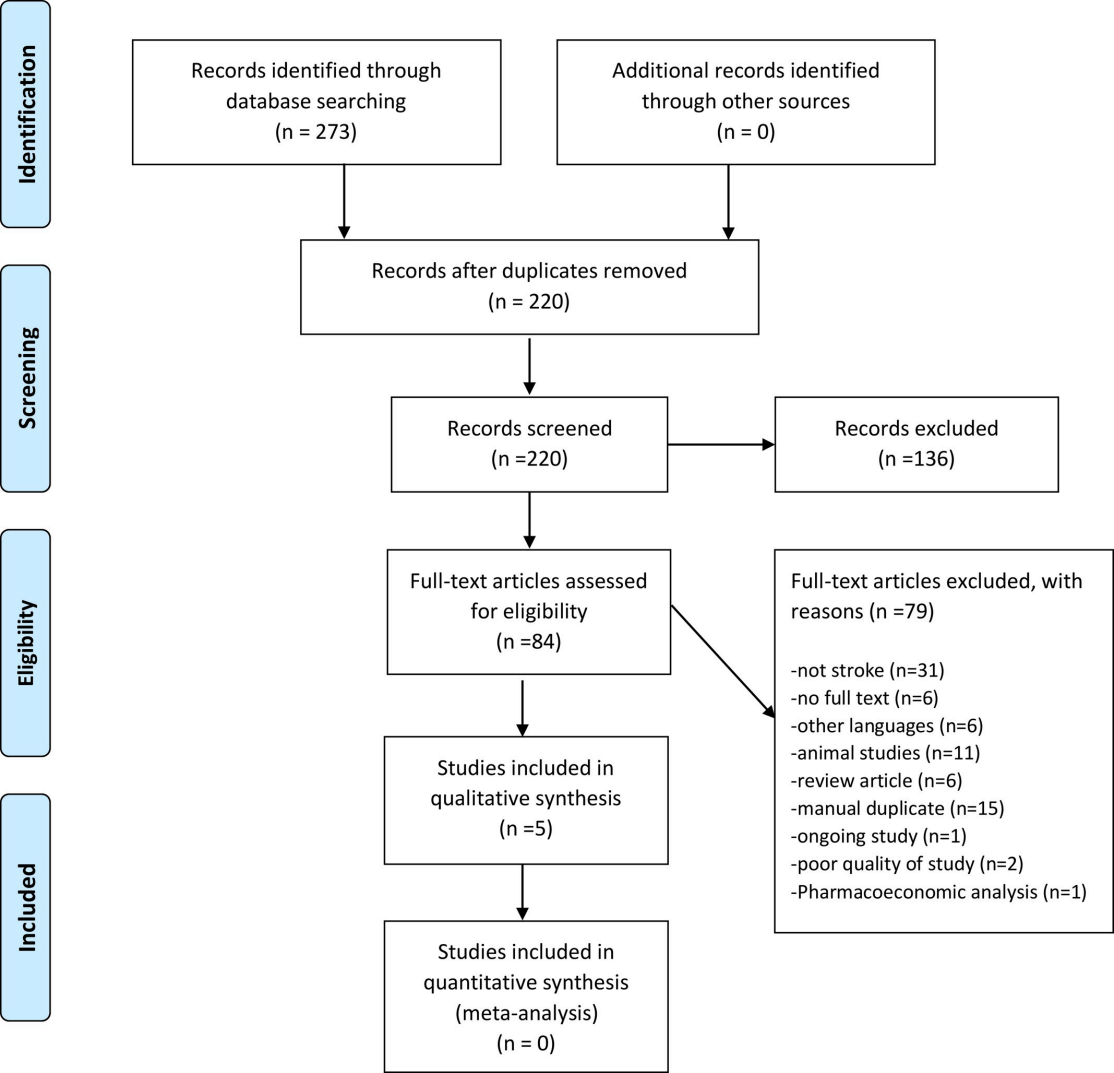
Selection of studies for inclusion/exclusion.

The electronic literature search identified 273 unique citations. The total number of citations after exclusion of duplicates was 220. Initial screening of titles and abstracts identified 84 citations that were considered eligible for full text review. After screening of full-text reports, 5 were retained and 79 were excluded. Fig 1 summarizes the process of study selection.

### Study characteristics

Five studies involving a total of 3879 patients were included (Table 1) [13,21–24]. Four studies used prospective data collection[13,21,23,24] and it was not clear if a fifth study was retrospective or prospective [22]. Four studies were non-randomized and open label [21–24] and one study was randomized and double-blinded [13]. The studies were published between 2007 and 2017. Four studies were published in Russian [21–24] and one study was published in English [13]. Planned study follow up time in four studies was between 30 days and 1 year [13,21,23,24] and follow up was not defined in one study [22]. Five studies enrolled patients with ischemic stroke [13,21–24] and one study also enrolled patients with hemorrhagic stroke [24]. Across the five included studies, 720 patients received Actovegin and where the treatment duration was described, the range was 10–180 days [13,21,23]. Two studies did not describe the treatment duration [22,24]. Two studies described the dose of Actovegin (250 ml of 20% solution IV daily for 10 days; 2 g/day IV for up to 20 days, followed by 1.2g/day PO for 6 days) [13,23] whereas three studies did not describe the amount of Actovegin administered [21,22,24]. Five studies allowed comparisons of Actovegin to standard stroke care or placebo [13,22–24] and one study allowed a comparison of Actovegin to piracetam [21]. The primary outcome was not defined in four studies [21–24] and in one study the primary outcome was change from baseline in ADAS-cog+ at 6 months [13].

**Table 1:** Characteristics of included studies.

Author (year); country; language	Study Design	Duration of follow up	N	Mean Age, years	Sex (male) n(%)	Population	Treatment Group	Control	Primary outcome	Summary of bias assessment
Observational Studies										
Ershov (2011) [22] Russia, Russian	Open-label, non-randomized, unclear if prospective or retrospective	Follow up length was not defined	1309	NR	173(56%) in treatment group (NR for control group)	Ischemic stroke (100%)	“Standard neuroprotective and reperfusion therapy” (details not defined) plus Actovegin (dose and route not defined) n = 309	Standard neuroprotective and reperfusion therapy (details not defined) n = 1000	Mortality based on a model that included the Gusev-Skvortcova scale, dose, and death rates	High risk of bias. Unclear if retrospective or prospective methods were used. Insufficient information to assess external and internal validity.
Shamalov (2010) [23] Russia; Russian	Open-label, non-randomized, prospective	Planned: 30 days; Actual: 30 days	104	65(±12)	67 (64%)	Ischemic stroke (100%)	All groups received “standard treatment” (not defined) plus: Citicoline 1000 mg IV daily x 10 days; N = 25 Actovegin 250 ml of 20% IV daily x 10 days; N = 26 Citicoline 1000 mg IV daily x 10 days plus Actovegin 250 ml of 20% IV daily x 10 days; N = 28	“Standard treatment” x 10 days (not defined), n = 25	Modified Rankin scale, Barthel Index, NIHSS (primary outcome not specified)	High risk of bias. Lack of reliable and consistent statistical analysis of outcomes.
Skoromets (2007) [24] Russia, Russian	Prospective non-randomized with matched controls	Planned: 1 year; actualfollow-up NR	1920	Ischemic stroke patients: 62.3 (min 36; max.80) Hemorrhagic stroke patients: 58.8 (min.33; max.76)	906 (47%)	Ischemic stroke (N = 1520, 19 groups of 80 patients) Hemorrhagic stroke (N = 400, 16 groups of 25 patients) Treatment and regimens were not clearly defined for the subgroups	“Standard treatment” (details not defined) + Actovegin (dose and route not defined) Patients with ischemic stroke, n = 80, Patients with hemorrhagic stroke n = 25	“Standard treatment” (details not defined) Patients with ischemic stroke, n = 80, Patients with hemorrhagic stroke n = 25 Controls were matched with treatment group based on age, sex, severity of stroke, disability	Composite of 3 scales: 1. Barthel physical function and activities of daily living, 2. Lindmark scale—physical 3. Scandinavian scale- and physical, verbal, time/space orientation, personal awareness	High risk of bias. Insufficient information provided about the study design, characteristics of the patient population, methods of assessment and validity of the primary outcome
Derev’yannykh (2007)[21] Russia, English	Open-label, non-randomized, prospective	Planned and actual: 30 days	43	56 (±2)	25 (58%)	Mild to moderate ischemic stroke (100%)	n = 32 Standard treatment plus Actovegin IV daily x 10 days (dose not specified), then 200 mg PO x 30 days	n = 11 Standard treatment plus piracetam IV daily x 10 days (dose not specified), then 1600 mg/day PO x 30 days	Gusev-Skvortsova scale, MMSE, EEG, (primary outcome not specified)	High risk of bias. Insufficient information provided about the characteristics of the patient population and methods of assessment.
Randomized Clinical Trials										
Guekht (2017) [13] Russia, Belarus, Kazakhstan; English	Parallel-group, randomized, multicenter, double-blind	1 year	503	70(±7)	241 (48%)	Acute supratentorial ischemic stroke (100%)	n = 248 Standard care (included antiplatelet agents and rehabilitation) plus Actovegin IV 2000 mg/day for up to 20 days, then 1200 mg/day PO x 6 months	n = 255 Standard care plus placebo	Change from baseline in ADAS-cog+ at 6 months.	Some concerns.

NR = not reported; SD = standard deviation

The results of the studies are shown in Table 2 and the quality assessment results are summarized in Tables 3–5. A pooled analysis was not performed due to clinically relevant differences in study design, dissimilarities in outcomes between the studies and heterogeneity of study population.

**Table 2:** Outcomes and results of the included studies.

		Results				
Author, year, country; language of publication	Primary Outcome	Cognition	Neurologic Symptoms	Activities of Daily Living, or Disability	Adverse Events	Authors’ Conclusions
Observational Studies						
Ershov[22] 2011 Russia; Russian	Mortality based on a model that included the Gusev-Skvortsova scale, dose, and death rates	No data on cognition, neurologic symptoms or activities of daily living. A unvalidated model that included mortality and symptoms was used to predict risk of death after stroke. Data did not allow a clear interpretation of the impact of Actovegin compared to controls.			Deaths, n/N(%) Actovegin group: 46/309(14.9%) Control group: 188/1000(18.8%) No information on length of observation Adverse events NR	The risk of death is decreased as the dose of Actovegin is increased (dose information not stated).
Shamalov et.al. [23] 2010 Russia; Russian	Not specified	- NIHSS scores, Day 10 Actovegin, n = 26: 6.2 Standard treatment, n = 25: 6.9; p>0.05 Day 30 Actovegin, n = 26: 6.2 Standard treatment, n = 25: 6.9; p>0.05 Barthel Index* Day 30 Actovegin, n = 26: 87.7(±13.4) Standard treatment, n = 25 67.9(±26.5); p<0.05 Rankin* 0 or 1 score at Day 30 Actovegin: 12/26(46.2%) Standard treatment: 4/25(16.0%)			Deaths, n/N(%) Actovegin: 0/26(0) Standard treatment: 1/25(4%) Adverse events NR	No conclusions provided regarding the effectiveness of Actovegin versus standard treatment
Skoromets et.al. [24] 2007 Russia; Russian	“Recovery of Function” as measured by a composite of 3 scales: 1. Barthel physical function and activities of daily living, 2. Lindmark scale—physical 3. Scandinavian scale- and physical, verbal, time/space orientation, personal awareness	No data were presented separately for the Barthel, Lindmark or Scandinavian scales. “Recovery of function” in patients with ischemic stroke, Taking Actovegin, n = 80: 78.3% Not taking Actovegin, n = 80: 24.8% “Recovery of function” in patients with hemorrhagic stroke, Taking Actovegin, n = 25: 82.5% Not taking Actovegin, n = 25: 47.8%			Adverse events NR	Actovegin is more ‘effective’ than no treatment based on a composite score system (no information provided on validity of composite score system)
Derev’yannykh et.al. [21] 2007 Russia; English	Not specified	MMSE* Day 10 Actovegin n = 32: 24.1(±0.14) Piracetam n = 11: 23.2(±0.11); p>0.05 Day 30 Actovegin n = 32: 29.1(±0.16) Piracetam n = 11: 25.9(±0.24); p<0.05 Gusev-Skvortsova ischemic scale* Day 30 Actovegin n = 32: 45.1(±0.24) Piracetam n = 11: 42.1(±0.15); p<0.05			No deaths. Allergic reaction to Actovegin, n = 1 (3%) ‘Significant increases in the rheographic index’ for Actovegin compared to Piracetam	- Actovegin has ‘great potential’ for the treatment of patients with mild-moderate ischemic stroke, including for the correction of cognitive disorders.
Randomized Clinical Trials						
Guekht et.al. [13] 2017 Russia, Belarus, and Kazakhstan; English	Change from baseline in ADAS-cog+ at 6 months.	ADAS-cog+ CFB Month 6 (SE) Actovegin (N = 248) -6.8(0.6) Placebo (N = 255) -4.6(0.6) MDC -2.3(-3.9, -0.7); p = 0.005 Responders (≥4 points increase) Actovegin: 130/208 (62.5%) Placebo: 113/216 (52.3%) Difference: 10.2% (95%CI: 0.8–19.5); p = 0.034 Dementia diagnosis Month 6 Actovegin: 16/218 (7.3%) Placebo: 24/228 (10.5%) Difference: -3.2% (95%CI: -8.5, 2.1); p = 0.251	NIHSS CFB Month 6 (SE) Actovegin: -3.2(0.1) Placebo: -3.2(0.1) Difference: 0.0 (95%CI -0.3, 0.2); p = 0.89	Barthel Index Median score was 100 for both groups at months 3 and 6. EuroQol EQ-5D and Beck Depression Inventory-II scores “showed similar responses in both groups” at months 6 and 12	Adverse events Actovegin: 89/250 (35.6%) Placebo: 96/253 (37.9%) SAE Actovegin: 22/250 (8.8%) Placebo: 17/253 (6.7%) WDAE Actovegin: 21/250 (8.4%) Placebo: 12/253 (4.7%) Recurrent Events: Ischemic Stroke/TIA Actovegin: 13(5.2%) Placebo: 7(2.8%) Intracerebral hemorrhage Actovegin: 1(0.4%) Placebo: 0	“Actovegin had a beneficial effect on cognitive outcomes in patients with poststroke cognitive impairment. The safety experience was consistent with the known safety and tolerability profile of the drug. These results warrant confirmation in additional robustly designed studies.”

Note: Data are mean (standard error) or n/N(%) unless otherwise stated.

ADAS-cog+ = Alzheimer’s Disease Assessment Scale, cognitive subscale extended version; AE = adverse event; CFB = change from baseline (least squares); MDC = mean difference of change from baseline; MMSE = Mini Mental Status Exam; NIHSS = National Institutes of Health Stroke Scale; SAE = serious adverse event; SE = standard error; WDAE = withdrawal due to adverse event.

*Baseline data not reported by treatment.

**Table 3:** Newcastle Ottawa Risk of Bias Assessment of Observational Studies–cohort studies.

Study	Representativeness of the intervention cohort	Selection of the non intervention cohort	Ascertainment of intervention	Demonstration of outcome of interest not present initially	Comparability of cohorts	Assessment of outcome	Follow up long enough	Adequacy of cohort follow up
**Ershov [22] 2011 Russia**	No description of the derivation of the cohort	No description of the derivation of the cohort	No description	Yes	No description of baseline characteristics. Analysis did not control for confounding factors.	No description of outcome assessment methods	No information provided	The number of patients lost-to-follow up is not reported
**Shamalov [23]** **2010** **Russia**	Somewhat representative (inpatients from one center)	Drawn from the same group of people as intervention	No description	Yes	Controlled for time from infarct to treatment	No description of outcome assessment methods	Yes	Not stated
**Skoromets [24]** **2007 Russia**	Somewhat representative (inpatients from one center)	Drawn from the same community(Matched- controls)	No description	Yes	Matched controls were used but no baseline characteristics were provided	No description of source or methods for outcome assessment	Yes	Not stated
**Derev’yannykh [21]** **2007** **Russia**	Somewhat representative (mild/moderate stroke, inpatients from one center)	Drawn from the same community as intervention	No description	Yes	Baseline scores not provided by separate treatment group	No description of outcome assessment methods	Yes	Not stated

**Table 4:** Risk of bias assessment for the included randomized controlled trials.

Domain	Support for judgment	Review authors’ judgment	Overall Study Risk
**Guekt et al [13]**			Some concerns
**Selection bias**			
Random sequence generation	Patients were centrally randomized via a computerized system in blocks of 4	Low	
Allocation concealment	The randomization sequence was centrally computer generated.	Low	
**Performance bias**			
Blinding of participants and personnel	During double-blind treatment and until end of follow-up, all investigators and patients were masked to treatment assignment.	Low	
**Detection bias**			
Blinding of outcome assessment	Details about the similarity of Actovegin IV/PO and placebo IV/PO were not provided. Some outcomes (adas-cog) could have been biased if investigators or patients could tell the difference between Actovegin and placebo.	unclear	
**Attrition bias**			
Incomplete outcome data	15% attrition in Actovegin arm, and 13% in the placebo arm.	Low	
**Reporting bias**			
Selective reporting	Outcomes established in the study protocol on clinicaltrials.gov appear to be reported in the publication	Low	
**Other bias**			
Other sources of bias	Study was funded by Takeda Pharmaceuticals, the manufacturer of Actovegin. 4 of 5 authors worked as consultants for Takeda or were employees of Takeda. It was not clear to what degree Takeda was involved in the analysis and reporting of the results.	unclear	
**Outcomes**	Alzheimer’s Disease Assessment Scale + Cognitive subscale extended version, Montreal Cognitive Assessment Scale, dementia diagnosis, National Institutes of Health Stroke Scale, Barthel Index, EuroQoL EQ-5D, Beck Depression Inventory		

**Table 5:** Quality assessment of the included randomized trials using the Jadad scale.

Study	Study described as randomized? Adequate details provided?	Study described as double–blinded? Adequate detail provided?	Withdrawals appropriately accounted for?	Allocation concealment considered adequate, inadequate
Guekht 2017 [13]	Yes/ Yes	Yes/ Yes	Yes	Adequate

In 2011, Ershov et al reported an open-label non-randomized study of patients with ischemic stroke receiving Actovegin (N = 309) or standard therapy (N = 1000) [22]. Standard therapy was described as ‘neuroprotective and perfusion’ therapy but the components of standard therapy were not described. The dose and regimen used for Actovegin was not reported. It was unclear whether the data were collected in retrospect or if patients were enrolled and the data were collected prospectively. The primary outcome was mortality and the crude mortality rate was 14.9% in the group receiving Actovegin and 18.8% in the group receiving standard therapy. Other outcome data were collected using the Gusev-Skvortsova scale, which measures neurologic deficit, but these data were not presented in a way as to allow a clear comparison between patients taking Actovegin and patients in the control group. Risk of bias assessment showed a high risk of bias for this study. The study report lacked a clear description of the population and prevalence of important prognostic factors, methods for outcome assessment and there was very little description regarding the follow up of study subjects.

In 2010, Shamalov and colleagues compared Actovegin to standard treatment in a prospective, non-randomized cohort with ischemic stroke (N = 104). The data from a single center showed no statistically significant differences between Actovegin and standard treatment at day 10 and day 30. The authors reported statistically significant improvements in the Barthel Index at day 30 favoring Actovegin (87.7±13.4; n = 26) compared to standard treatment (67.9±26.5; n = 25; p<0.05). There was one death in the standard treatment group. The lack of randomization in this study resulted in an assessment of high risk of bias.

In 2007, Skoromets et al reported a prospective non-randomized study using matched controls. They reported using a composite outcome that included parts of the Barthel, Lindmark and Scandinavian scales [24]. Controls were matched based on several prognostic factors, but insufficient information was provided to examine the balance of prognostic factors between the treatment and control groups. Authors reported a higher proportion of patients had recovery of function after ischemic stroke in the Actovegin group (78.3%, n = 80) compared to patients who received standard treatment without Actovegin (24.8%, n = 80). The validity of the composite outcome the timing of its evaluation was not clear and risk of bias was assessed as high. The study enrolled matched controls but lacked details regarding baseline characteristics and there was no description of methods for outcome assessment.

In 2007, Derev’yannykh et al assessed the efficacy of standard treatment plus Actovegin relative to standard treatment plus piracetam in 43 patients with mild to moderate ischemic stroke. The choice of piracetam as a comparator limits external validity of this study because piracetam is not part of standard stroke care in many countries. The primary outcome was not specified. Statistically significant differences in the Mini Mental Status Exam and the Gusev-Skvortsova scale were reported in favour of Actovegin relative to piracetam at day 30, but baseline values were not reported. One allergic reaction to Actovegin was observed. Insufficient data were provided regarding the baseline characteristics of the patients resulting in an assessment of high risk of bias.

In 2017, Guekht and colleagues performed a randomized, placebo-controlled trial in which investigators and patients were blinded to treatment assignment. The primary outcome was change from baseline of the ADAS-cog+. At 6 months, the ADAS-cog+ change from baseline was -6.8 for Actovegin and -4.6 for placebo (mean difference of change -2.3 [95%CI: -3.9, -0.7; p = 0.005]). There was no statistically significant difference between Actovegin and placebo in the proportion of patients diagnosed with dementia by month 6. The results of the NIHSS at month 6 showed no statistically significant difference between Actovegin and placebo groups. There were no marked differences between treatment groups in quality of life as measured by the EuroQol EQ-5D. There were 14 (5.6%) recurrent stroke events in patients taking Actovegin (13 ischemic stroke or transient ischemic attack, 1 intracerebral hemorrhage) and 7(2.8%) in patients taking placebo (odds ratio 2.09[95%CI 0.83,5.26] p = 0.124). The proportion of patients discontinuing the study treatment due to adverse events was higher in the Actovegin group (21[8.4%]) compared to the placebo group (12[4.7%]). There were 7(2.8%) deaths in the Actovegin group and 6(2.4%) deaths in the placebo group.

## Discussion

### Trials

To our knowledge, this is the first systematic review of the primary literature to focus on the clinical effects of Actovegin in patients with ischemic stroke. While clinicians in some regions may be unfamiliar with Actovegin, it is used in East Asia, Eastern Europe, and Central Asia. It is commonly referred to as a “neuroprotector” and has been frequently used to treat patients with ischemic stroke in Russia [25,26]. Pharmaceutical sales data suggest that usage is widespread, though declining, as Actovegin has frequently appeared on the top-15 drugs by sales volume, in every country of the Commonwealth of Independent States (CIS) for years 2018–2020 [27]. This includes usage through government insurance schemes [28] and private purchase. The absolute extent to which Actovegin is used in the post-stroke context is not known.

A strength of our review is that our research team included native Russian speakers and this allowed inclusion of Russian language publications. This is relevant since a significant proportion of the research and clinical usage of Actovegin has occurred in countries in which Russian is spoken. Additionally, a comprehensive literature search was undertaken and standard systematic review methodology was used in accordance with PRISMA guidance [29].

One of the limitations of our review is that some studies that were published in languages other than Russian or English were excluded which may be relevant. However, the main limitation of our review was the dearth of high-quality studies upon which to base conclusions about Actovegin in patients who have suffered an ischemic stroke. Given the widespread use of Actovegin in many countries, it was surprising to find so few prospectively conducted clinical trials evaluating its effectiveness. The connection between evidence and practice patterns in Central Asia has not been well studied and is therefore poorly understood; it is possible that recent declines in Actovegin usage may be related to a growing regional awareness of the low volume and quality of evidence. Because of lack of randomized controlled trials, an attempt was made to expand our evidence base by including observational studies with a control group. In four non-randomized studies, risk of bias was assessed using the Newcastle-Ottawa scale; all studies scored poorly, failing to score well because of lack of clear reporting of methods, unclear statistical methods and lack of evidence that prognostic factors were well balanced between the treatment groups. One randomized controlled trial was included in this review. Quality assessment of this trial produced a Jadaad score indicating moderate-high quality and low risk of bias for most domains of the Cochrane Risk of Bias Tool. Meta-analysis of data was not possible due to the clinical heterogeneity of study designs and different outcomes measured.

The typical cost of one month’s supply of Actovegin is high relative to the median household income in the regions where it is sold. For example, the mean salary in Kazakhstan, a country in which Actovegin has been used extensively, is 572 US dollars per month [30]. One month supply of Actovegin represents approximately 4.4% ($25) of the average income. A consideration and area for future research is to incorporate the uncertainties from the clinical data into pharmacoeconomic analyses [26].

### Efficacy

This systematic review was performed to identify evidence for Actovegin in the post-stroke context and its impact on clinically relevant and validated outcomes. No consistent evidence on improved survival, quality of life, neurologic symptoms, activities of daily living or disability was identified in this systematic review. It is difficult to make inferences from observational studies whose populations were not well described with respect to prognostic risk factor distribution and outcome measurement. One study [13] showed improvements in the primary outcome, ADAS-cog+, for Actovegin compared with placebo at 6 months. There are limitations to the finding of this study; the validity of the ADAS-cog+ scale in the Russian language and in the post-stroke context is not clear. The clinical impact of the change in ADAS-cog+ that was found in the study is also unclear. The minimal clinically important difference (MCID) cited by the authors was 4 in the setting of mild to moderate Alzheimer’s Dementia. The primary outcome of the study demonstrated a least squared mean difference of -2.3 in the patients who received Actovegin compared to those who received placebo. While this finding was statistically significant it is unclear if this difference is clinically relevant as the MCID of the ADAS-cog+ in patients with cognitive impairment after an ischemic stroke was not reported by the authors. Concerns regarding the ability of the ADAS-cog+ to detect pre-dementia cognitive impairment have been raised in the medical literature [31]. In general, the ADAS-cog+ is used as a research tool rather than tool for diagnosing or following patients with cognitive impairment after an ischemic stroke. Furthermore, the author’s primary endpoint analysis of the ADAS-cog+ at month 6 did not include 10% of the patients who were originally randomized in the study. Taken together, the clinical importance of an improvement in the ADAS-cog+ to patients is uncertain. Translation of the evidence in this systematic review into clinical practice would involve a comparison of the level of evidence that exists for well-established pharmacologic interventions. The quality assessment suggests that the quality of evidence is not as high as that which exists for thrombolytic and antiplatelet medications (e.g. alteplase, aspirin, clopidogrel).

### Adverse events

Despite its widespread usage in many countries over several decades, the safety of Actovegin has not been extensively evaluated in prospective studies with a control group and long-term follow-up. Of the five studies in this systematic review, Guekht et al was designed to prospectively collect data on adverse events with Actovegin compared to placebo. Importantly, the authors reported a numerically higher rate of discontinuation due to adverse events. They reported a higher incidence of recurrent ischemic stroke, transient ischemic attack or intracerebral hemorrhage in patients taking Actovegin compared to placebo [13]. Details regarding the patients who suffered a subsequent stroke were not reported. Severity and outcome of the secondary strokes, the time of their occurrence and any other risk factors that may have been present in these patients could illuminate possible causes. These findings, and a difference of this magnitude in an outcome of significant clinical importance is worthy of further research.

## Conclusions

This systematic review identified four low quality studies and one moderate-high quality study evaluating the role of Actovegin in the care of adult patients after an ischemic stroke. A narrative review of these trials did not identify significant improvement in mortality or morbidity with the use of Actovegin. The currently available data demonstrate that the benefits are uncertain and that there is potential risk of harm in patients with stroke. More evidence is needed from rigorously designed clinical trials to justify the role of Actovegin in patients with ischemic stroke.

## Supporting information

S1 Table Literature Search Strategy. (PDF)

S2 Table PRISMA 2020 for Abstract Checklist. (DOCX)

S3 Table PRISMA 2020 Checklist. (DOCX)
