# Supplementary material for: Actovegin in the management of patients after ischemic stroke: A systematic review
Source: PLoS One. 2022 Jun 30;17(6):e0270497. doi: 10.1371/journal.pone.0270497 (PMC9246213; doi:10.1371/journal.pone.0270497)
Supplement: S1 Table — (PDF) [file pone.0270497.s001.pdf]

Supplemental Material: Literature Search Strategy

Database	Search strategy	Number of studies
Cochrane	1) (((Protein-free):ti,ab,kw OR (Deprotein*):ti,ab,kw OR (antigen-free):ti,ab,kw OR (extract):ti,ab,kw)) AND (("calf blood"):ti,ab,kw OR ("calves blood"):ti,ab,kw))) OR (((calf):ti,ab,kw OR (calves):ti,ab,kw)) AND ((Hemoderivat*):ti,ab,kw OR (hemodialysate):ti,ab,kw OR (haemoderivat*):ti,ab,kw OR (haemodialysate):ti,ab,kw)) OR (((actihamyl):ti,ab,kw OR (actihemyl):ti,ab,kw)) OR ((Actihaemyl):ti,ab,kw OR (Actihaemyin):ti,ab,kw)) OR (("actovegin"):ti,ab,kw OR (Solcoseryl):ti,ab,kw OR (Solceseryl):ti,ab,kw OR (solcoceryl):ti,ab,kw OR (solkoseryl):ti,ab,kw))<br><br>2) (MeSH[Brain Ischemia] OR MeSH[Brain Infarction] OR [Carotid Artery Diseases] OR MeSH[Carotid Artery Thrombosis] OR MeSH[Carotid Artery, Internal, Dissection] OR MeSH[Intracranial Arterial Diseases] OR MeSH[Cerebral Arterial Diseases] OR MeSH[Infarction, Anterior Cerebral Artery] OR MeSH[Infarction, Middle Cerebral Artery] OR MeSH[Infarction, Posterior Cerebral Artery] OR MeSH[Intracranial Embolism and Thrombosis] OR MeSH[Stroke] OR MeSH: [Basal Ganglia Cerebrovascular Disease])<br><br>3) #2 AND #1<br>4) (brain OR cerebr* OR cerebell* OR vertebrobasil* OR hemispher* OR intracran* OR intracerebral OR infratentorial OR supratentorial OR middle next cerebr* OR mca* OR “anterior circulation” OR “basilar artery” OR “vertebral artery”) near/5 (stroke* OR isch*mi* OR infarct* OR thrombo* OR emboli* OR occlus* OR hypoxi*)<br>5) #4 AND #1<br>6) stroke OR isch*mi* OR "hemorrhagic stroke" OR "cerebrovascular accident" OR "cerebrovascular apoplexy" OR "brain vascular accident"<br>7) #6 AND #1<br>8) "Stroke rehabilitation" OR "stroke management" OR post-stroke OR "post-stroke complications" OR poststroke OR “Neurological Rehabilitation”<br>9) #8 AND #1<br>10) #3 OR #5 OR #7 OR #9	24
Medline (EBSCO)	1) (((Protein-free):ti,ab,kw OR (Deprotein*):ti,ab,kw OR (antigen-free):ti,ab,kw OR (extract):ti,ab,kw)) AND (("calf blood"):ti,ab,kw OR ("calves blood"):ti,ab,kw))) OR (((calf):ti,ab,kw OR (calves):ti,ab,kw)) AND ((Hemoderivat*):ti,ab,kw OR (hemodialysate):ti,ab,kw OR (haemoderivat*):ti,ab,kw OR (haemodialysate):ti,ab,kw)) OR (((actihamyl):ti,ab,kw OR (actihemyl):ti,ab,kw)) OR ((Actihaemyl):ti,ab,kw OR (Actihaemyin):ti,ab,kw)) OR (("actovegin"):ti,ab,kw OR (Solcoseryl):ti,ab,kw OR (Solceseryl):ti,ab,kw OR (solcoceryl):ti,ab,kw OR (solkoseryl):ti,ab,kw))<br>2) (MH "Brain Ischemia+") OR (MH "brain infarction+") OR (MH "brain infarction+") OR (MH "carotid artery, internal, dissection+") OR (MH "intracranial arterial diseases+") OR (MH "cerebral arterial diseases+") OR (MH "Infarction, Anterior Cerebral Artery") OR (MH "Infarction, Middle Cerebral Artery") OR (MH "Infarction, Posterior Cerebral Artery") OR (MH "Intracranial Embolism and Thrombosis+") OR (MH "Stroke+") OR (MH "Basal Ganglia Cerebrovascular Disease+") OR (MH "Vertebral Artery Dissection")<br>3) #1 AND #2<br>4) ((brain OR cerebr$ OR cerebell$ OR vertebrobasil$ OR hemispher$ OR intracran$ OR intracerebral OR infratentorial OR supratentorial OR middle next cerebr$ OR mca$ OR “anterior circulation” OR “basilar artery” OR “vertebral artery”) N5 (stroke$ OR isch?mi$ OR infarct$ OR thrombo$ OR emboli$ OR occlus$ OR hypoxi$))<br>5) #4 AND #1<br>6)stroke OR isch*mi$ OR "hemorrhagic stroke" OR "cerebrovascular accident" OR "cerebrovascular apoplexy" OR "brain vascular accident"<br>7) #6 AND #1<br>8) "Stroke rehabilitation" OR "stroke management" OR post-stroke OR "post-stroke complications" OR poststroke OR “Neurological Rehabilitation”<br>9) #1 AND #8<br>10) #3 OR #5 OR #7 OR #9	67

PubMed	1) ((((("calf blood"[All Fields] OR "calves blood"[All Fields]) AND (((Protein-free[All Fields] OR (deprotein[All Fields] OR deproteinace[All Fields] OR deproteinaci[All Fields] OR deproteinate[All Fields] OR deproteinated[All Fields] OR deproteinateda[All Fields] OR deproteinates[All Fields] OR deproteinating[All Fields] OR deproteination[All Fields] OR deproteinationultrafiltration[All Fields] OR deproteine[All Fields] OR deproteined[All Fields] OR deproteinezation[All Fields] OR deproteiniation[All Fields] OR deproteinisation[All Fields] OR deproteinise[All Fields] OR deproteinised[All Fields] OR deproteinisierte[All Fields] OR deproteinisiertem[All Fields] OR deproteinisierten[All Fields] OR deproteinisiertes[All Fields] OR deproteinising[All Fields] OR deproteinizant[All Fields] OR deproteinizate[All Fields] OR deproteinizated[All Fields] OR deproteinization[All Fields] OR deproteinizationed[All Fields] OR deproteinizations[All Fields] OR deproteinizatsii[All Fields] OR deproteinizatsiia[All Fields] OR deproteinizatsiiu[All Fields] OR deproteinizattion[All Fields] OR deproteinize[All Fields] OR deproteinized[All Fields] OR deproteinizer[All Fields] OR deproteinizes[All Fields] OR deproteinizied[All Fields] OR deproteinizing[All Fields] OR deproteinizirovannogo[All Fields] OR deproteinizirovannym[All Fields] OR deproteiniziruiushchei[All Fields] OR deproteinizovane[All Fields] OR deproteinizzanti[All Fields] OR deproteinizzata[All Fields] OR deproteinizzati[All Fields] OR deproteinizzato[All Fields] OR deproteinizzazione[All Fields] OR deproteinovaneho[All Fields] OR deproteinzated[All Fields] OR deproteinzation[All Fields] OR deproteinzed[All Fields])) OR antigen-free[All Fields]) OR extract[All Fields])) OR ((calf[All Fields] OR calves[All Fields]) AND ((((hemoderivate[All Fields] OR hemoderivates[All Fields] OR hemoderivative[All Fields] OR hemoderivatives[All Fields]) OR hemodialysate[All Fields]) OR (haemoderivat[All Fields] OR haemoderivate[All Fields] OR haemoderivaten[All Fields] OR haemoderivates[All Fields] OR haemoderivative[All Fields] OR haemoderivatives[All Fields])) OR haemodialysate[All Fields]))) OR ((((("actihaemyl"[MeSH Terms] OR "actihaemyl"[All Fields]) OR Actihaemyin[All Fields]) OR "S 1021"[All Fields]) OR "37239-28-4"[EC/RN Number]) OR "SS-094"[All Fields]) OR actihemyl[All Fields])) OR ("Actovegin"[Supplementary Concept] OR "Actovegin"[All Fields] OR "actovegin"[All Fields])) OR (("actihaemyl"[MeSH Terms] OR "actihaemyl"[All Fields] OR "solcoseryl"[All Fields]) OR solkoseryl[All Fields])<br>2) (((((("Brain Ischemia"[Mesh] OR "Brain Infarction"[Mesh]) OR "Carotid Artery Diseases"[Mesh]) OR "Carotid Artery Thrombosis"[Mesh]) OR "Carotid Artery, Internal, Dissection"[Mesh]) OR "Intracranial Arterial Diseases"[Mesh]) OR "Cerebral Arterial Diseases"[Mesh]) OR "Infarction, Anterior Cerebral Artery"[Mesh]) OR "Infarction, Middle Cerebral Artery"[Mesh]) OR "Infarction, Posterior Cerebral Artery"[Mesh]) OR "Intracranial Embolism and Thrombosis"[Mesh]) OR "Stroke"[Mesh]) OR "Basal Ganglia Cerebrovascular Disease"[Mesh]) OR "Vertebral Artery Dissection"[Mesh])))))<br>3) #1 AND #2<br>4) ((("Stroke rehabilitation"[All Fields] OR "post-stroke complications"[All Fields]) OR poststroke[All Fields]) OR "Neurological Rehabilitation"[All Fields]) OR "stroke management"[All Fields]) OR post-stroke[All Fields])))<br>5) #1 AND #4<br>6) ((brain OR cerebr* OR cerebell* OR vertebrobasil* OR hemispher* OR intracran* OR intracerebral OR infratentorial OR supratentorial OR middle next cerebr* OR mca* OR “anterior circulation” OR “basilar artery” OR “vertebral artery”) AND (stroke* OR isch?mi* OR infarct* OR thrombo* OR emboli* OR occlus* OR hypoxi*))<br>7) #1 AND #6<br>8) #3 OR #5 OR #7	84
EMBASE (Ovid)	1) ((("calf blood" or "calves blood").mp.) AND (Protein-free or Deprotein* or antigen-free or extract).mp.)) OR ((calf or calves).mp.AND (Hemoderivat* or hemodialysate or haemoderivat* or haemodialysate).mp.)) OR ((Actihaemyl or Actihaemyin or actihamyl or actihemyl').mp.) OR (actovegin.mp. or exp actovegin/) OR (Solcoseryl or 8070-90-6 or solceseryl or solcoceryl or solkoseryl).mp.)))<br>2) ((("brain ishemia" or "brain infarction" or "carotid artery disease").mp.) OR (cerebrovascular accident/) OR (ischemia/ or brain ischemia/) OR (brain hemorrhage/))<br>3) #1 AND #2<br>4) ((stroke rehabilitation/) OR (("stroke management" or post-stroke or "post-stroke complications").mp.))<br>5) #1 AND #4<br>6) #3 OR #5	94
clinicaltrials.gov	Condition: Stroke OR ischemia<br>Other terms: actovegin OR calf blood<br>Study type: all studies<br>Study results: all studies	5
